# Editorial: Engineering applications of neurocomputing, volume II

**DOI:** 10.3389/fnbot.2023.1306042

**Published:** 2023-10-23

**Authors:** Long Wang, Zhe Song, Zijun Zhang, Chao Huang

**Affiliations:** ^1^School of Computer and Communication Engineering, University of Science and Technology Beijing, Beijing, China; ^2^School of Business, Nanjing University, Nanjing, China; ^3^School of Data Science, City University of Hong Kong, Kowloon, Hong Kong SAR, China; ^4^State Key Laboratory of Internet of Things for Smart City, University of Macau, Macau SAR, China

**Keywords:** neurocomputing, engineering systems, neural networks, manufacturing, healthcare

Inspired by how the human brain works, neurocomputing algorithms, including deep learning, reinforcement learning, and neurodynamic optimization, have achieved tremendous success in various applications across many domains, e.g., visual object tracking, speech recognition, human-level control, text understanding, and real-time optimization.

Various types of intelligent equipment and hardware devices are developed to implement the neurocomputing models for engineering systems. Deep learning has been employed for industrial robotic applications, including stereo reconstruction, object pose recognition, and product quality check. With the advent of Internet of Things and edge computing devices, predictive maintenance of engineering equipment is gaining popularity using deep reinforcement learning. Embedded convolutional neural networks are widely utilized for autonomous vehicle control. The success of applying neurocomputing approaches and related hardware implementations in different engineering domains, such as intelligent manufacturing, energy internet and smart healthcare, has proved the potential of employing neurocomputing for solving real problems in various engineering fields.

Nowadays, advances in sensor and data storage technologies have enabled cumulation of a large amount of data from engineering systems. Driven by big data generated from engineering systems, neurocomputing and its hardware implementation will continually transform engineering systems to more intelligent forms.

This Research Topic aims to provide a forum for researchers to present the latest research on applications of neurocomputing algorithms and neurocomputing-based hardware in engineering systems. The Volume II of this Research Topic collected another 5 high quality papers reporting the latest applications of neurocomputing in wind energy, nuclear power, and autonomous driving.

The paper titled “*Wind speed interval prediction based on the hybrid ensemble model with biased convex cost function*” by Long et al. proposed a combination interval prediction based hybrid ensemble model for short-term wind speed prediction. The combination interval prediction model employed the extreme learning machine as the predictor with a biased convex cost function. With the benefit of the biased convex cost function and ensemble technique, the high computational efficiency and stable performance of the proposed prediction model was guaranteed simultaneously. The superiority of the proposed interval prediction model was verified based on comprehensive experiments.

The paper titled “*Neural network aided flexible joint optimization with design of experiment method for nuclear power plant inspection robot*” by Wang G. et al. developed a neural network aided flexible joint structure optimization method with the Design of Experiment method for the nuclear power plant inspection robot. With this method, the joint's dual-spiral flexible coupler was optimized regarding the minimum mean square error of the stiffness. The optimal flexible coupler was demonstrated and tested via experiments.

The paper titled “*Soft-masks guided faster region-based convolutional neural network for domain adaptation in wind turbine detection*” by Xu et al. reported a synthetic generation method trying to simulate the real wind turbines from the aspects of style and content. Meanwhile, a novel soft-masks guided faster region-based convolutional neural network was developed for domain adaptation in wind turbine detection. The effectiveness of the proposed approach was evaluated by comparing with other representative models on the real dataset.

The paper titled “*Real-time depth completion based on LiDAR-stereo for autonomous driving*” by Wei et al. introduced a real-time LiDAR-stereo depth completion network without 3D convolution to fuse point clouds and binocular images using injection guidance. At the same time, a kernel-connected spatial propagation network was utilized to refine the depth. The output of dense 3D information was more accurate for autonomous driving. Experimental results on the KITTI dataset showed that the proposed method used real-time techniques effectively.

The paper titled “*A semantic segmentation scheme for night driving improved by irregular convolution*” by Xuantao et al. investigated a fuzzy information complementation strategy based on generative models and a network that fuses different intermediate layer outputs to complement spatial semantics. The experimental results demonstrated that the proposed method can effectively cope with various problems faced by night driving and enhance the model's perception.

This topic reported various applications of neurocomputing in the engineering domain. These neurocomputing-based approaches demonstrate great potential in solving real problems. With the innovative improvements from the algorithmic side, more successful applications of neurocomputing will appear in future engineering systems.

## Author contributions

LW: Writing—original draft. ZS: Writing—review and editing. ZZ: Writing—review and editing. CH: Writing—review and editing.

